# Orthodontic Facial Orthomorphia — Importance of Differentiation in Cases of Apparent Mandibular Protrusion

**Published:** 1904-03

**Authors:** William Ernest Walker

**Affiliations:** New Orleans


### ORTHODONTIC FACIAL ORTHOMORPHIA—IMPORTANCE OF DIFFERENTIATION IN CASES OF APPARENT MANDIBULAR PROTRUSION.[1]

BY WILLIAM ERNEST WALKER, D.D.S., M.D., NEW ORLEANS.

QUITE a little has been written on the etiology and treatment of cases presenting the appearance of protrusion of the mandible, the "chin-cap" treatment being recommended in young subjects and the removal of sections from each side of the mandible in older patients, and of late much has been written regarding the technic of this latter operation.

In a recent article Dr. E. H. Angle mentions a case in which this operation was successfully done in St. Louis,[2] and tells of a failure having been made in New Orleans.[3] I have heard rumors

---

[1] Read at the annual session of the American Medical Association, Section on Stomatology.

[2] Dental Cosmos, April, 1903.

[3] Items of Interest, April, 1903.

in New Orleans concerning this failure, but have never seen the case.

Much has been written about treatment and a little about etiology, but apparently very little thought has been given to diagnosis, and the object of this brief paper is to emphasize the importance of a correct diagnosis on which to base an intelligent prescription.

It appears to have been taken for granted that the case which has the appearance to the surgeon of being a case of mandibular protrusion must necessarily be such, but I think that by presenting a case illustrating the erroneousness of that assumption, I can make it evident that this is not a fact.

From boyhood this patient was in the hands of a prominent dentist, who more than once was asked if anything could be done to remedy the defect; the invariable reply being "No, except in case the patient be willing to undergo a surgical operation for the removal of a section from each side of the mandible." The patient, at the age of nineteen, called on me, and my first impression was, as his brother had previously told me, that his lower jaw was very prominent.

Study of the case, however, convinced me that it was very much less a case of mandibular protrusion and very much more a case of arrest of the superior maxillæ.

Without explaining the diagnosis to the patient, he was allowed to return to his dentist, to whom he stated that Dr. Walker had told him that his case was remediable without a surgical operation, and again the dentist expressed as his opinion that nothing could be done for him except the operation previously mentioned, and that if Dr. Walker could otherwise remedy the defect, and if the patient had confidence, a trial might be made.

I mention these details to emphasize the perfect satisfaction the dentist manifested in his own diagnosis and prescription, notwithstanding its erroneousness, and to thus emphasize the necessity for the word of caution I am uttering, in order that errors in diagnosis may be avoided, for, on the diagnosis the treatment depends, which makes for success or failure.

The patient has been taking a rest for some months, waiting for the alveolar process to redevelop around the teeth in their new position, which has now taken place and we are about to carry the upper teeth a step farther. This waiting was considered advisable in order not to carry the teeth too far away from the bone, and because it has been found that if these periods of rest be given, the teeth can be moved great distances, the alveolar process developing around the teeth in their new position just as it did originally, and as it does after fractures, provided that the teeth are held still.

We know that in some pathologic conditions in adult life there is a great difference in the ability of the alveolar process to reproduce itself, but the movements of the teeth in alternating periods of activity and rest so closely resemble physiologic processes that Nature assists us by placing energetic osteoblasts at our service.

In the present case, the space made by the moving forward of the bicuspids and oral teeth has been closed by the moving forward of the second molars, assisted by the developing of the third molars, for which room has thus been made.

The next step will be to apply an apparatus which I am now constructing and which will have the effect of moving the maxillary teeth forward, using the chin and forehead as anchorage. This apparatus will somewhat resemble a baseball mask, but will have to be worn only during the night, the molars affording anchorage to retain during the day what we have gained during the night.

I hope by limiting myself to this one phase of the subject to so focus your attention on the subject of differentiation as to give it sufficient emphasis to attract the attention of the profession to its importance.

