# Correction of Article in March Number

**Published:** 1904-04

**Authors:** L. C. Taylor


### CORRECTION OF ARTICLE IN MARCH NUMBER.

I desire to enter a protest against what will be, to many, misleading regarding Dr. Riggs's methods and treatment. On page 197, March issue of this journal, the writer states: "Dr. Riggs's surgical method was to scrape the surface of the root of the affected tooth to remove all salivary calculi, and also removing any necrosed bone."

Thus far the writer has stated the exact truth regarding Dr. Riggs's operation. Had he stopped here—but he adds: "then continuing with the cauterizing. Even extreme methods, as replantation, were resorted to where there was elongation of the incisor teeth."

This last part is entirely contrary to Dr. Riggs's practice or teachings. Dr. Riggs condemned cauterizing or excision of the festoons severely, as well as replantation. Dr. Riggs did not believe in therapeutics of any kind in connection with his treatment, depending wholly on the thoroughness of his operation; but occasionally, when patients felt they must have some wash, he would tell them, in apparent disgust, "Use a little tincture of myrrh, then."

L. C. Taylor.

